# Negative-index gratings formed by femtosecond laser overexposure and thermal regeneration

**DOI:** 10.1038/srep23379

**Published:** 2016-03-16

**Authors:** Jun He, Yiping Wang, Changrui Liao, Chao Wang, Shen Liu, Kaiming Yang, Ying Wang, Xiaocong Yuan, Guo Ping Wang, Wenjing Zhang

**Affiliations:** 1 Key Laboratory of Optoelectronic Devices and Systems of Ministry of Education and Guangdong Province, College of Optoelectronic Engineering, Shenzhen University, Shenzhen 518060, China; 2 Department of Electrical Engineering, The Hong Kong Polytechnic University, Hong Kong, China; 3 College of Electronic Science and Technology, Shenzhen University, Shenzhen 518060, China

## Abstract

We demonstrate a method for the preparation of negative-index fibre Bragg gratings (FBGs) using 800 nm femtosecond laser overexposure and thermal regeneration. A positive-index type I-IR FBG was first inscribed in H_2_-free single-mode fibre using a femtosecond laser directed through a phase mask, and then a highly polarization dependant phase-shifted FBG (P-PSFBG) was fabricated from the type I-IR FBG by overexposure to the femtosecond laser. Subsequently, the P-PSFBG was thermally annealed at 800 °C for 12 hours. Grating regeneration was observed during thermal annealing, and a negative-index FBG was finally obtained with a high reflectivity of 99.22%, an ultra-low insertion loss of 0.08 dB, a blueshift of 0.83 nm in the Bragg wavelength, and an operating temperature of up to 1000 °C for more than 10 hours. Further annealing tests showed that the thermal stability of the negative-index FBG was lower than that of a type II-IR FBG, but much higher than that of a type I-IR FBG. Moreover, the formation of such a negative-index grating may result from thermally regenerated type IIA photosensitivity.

Fibre Bragg gratings (FBGs) that can operate at high temperatures are critical in applications using high-power fibre lasers^1^,^2^,^3^,^4^,^5^ and fibre-optic sensors in harsh environments^3^,^4^,^5^,^6^,^7^,^8^,^9^. Fibre lasers are widely used for cutting and welding due to their high efficiency and excellent beam quality. Writing FBGs directly into the active fibre cores is a convenient way to create compact, monolithic, and low-loss laser resonator cavities. In the case of high-power fibre lasers, for example, Yb^3+^-doped fibre lasers with an output power of up to 50 kW are commercially available^1^. Thermally stable FBGs are required to endure such high-intensity optical field^2^. Furthermore, FBGs are also widely used as sensors due to the advantages of compact size, immunity to electromagnetic interference, and capability of large-scale multiplexing and remote interrogation. However, sensing in harsh environments, such as the oil and gas industries, power stations, aircraft engines and furnaces, always requires high operating temperatures from 400 °C to above 1000 °C^3^,^8^,^9^.

Traditional type I FBGs with positive index modulation are based on colour centre formation, and are only suited for operating temperatures below 450 °C^3^. Several types of FBG have been developed for use at increased operating temperatures. For example, type II FBGs are created using high-intensity UV pulses near the damage threshold of the fibre core and can withstand temperatures up to 1000 °C. However, type II gratings always have poor spectral shapes and relatively larger insertion loss^3^. Type IIA FBGs with negative index modulation are inscribed in H_2_-free fibres by UV overexposure^10^,^11^,^12^,^13^,^14^,^15^,^16^ or thermal regeneration^17^,^18^. The formation of type IIA FBGs is associated with reduced axial stress, and these gratings can withstand temperatures up to 800 °C^13^,^14^. Regenerated FBGs are created by thermal treatment of the original UV-inscribed type I gratings in H_2_-loaded fibres^3^,^8^,^9^,^19^,^20^,^21^,^22^,^23^. When the original type I FBG is heated to 800–1000 °C, there will be an erasure of the grating, followed by a progressive regeneration. The formation of regenerated FBGs is achieved through a glass structural transformation arising from the relaxation of a relatively high internal stress between the fibre core and cladding; these gratings can withstand temperatures up to 1200 °C^19^. However, the refractive index modulation induced by thermal regeneration is so weak that strong seed gratings are always required to obtain an adequate reflectivity in the regenerated FBGs^19^,^20^,^21^,^22^,^23^. For example, Canning *et al.* reported on a regenerated FBG with a high reflectivity of up to 98.42% by using an ultra-strong seed FBG with a transmission depth of more than 50 dB and a grating length of 50 mm^23^. Nevertheless, the reflectivity of common regenerated gratings with a grating length of several millimetres was typically less than 30%^19^,^20^,^21^,^22^.

The use of NIR femtosecond lasers has also been explored for inscribing FBGs with enhanced thermal stability^24^,^25^,^26^,^27^,^28^,^29^,^30^,^31^,^32^,^33^. Type I-IR gratings with positive index modulation are inscribed with low-intensity pulses, and can withstand temperatures below 500 °C^25^,^29^,^30^. Type II-IR gratings with structural changes in the fibre core are inscribed using high-intensity pulses, and can withstand temperatures above 1000 °C^24^,^25^,^26^,^27^,^28^,^29^,^30^,^31^,^32^,^33^. The operating temperatures of type II-IR gratings can be further increased up to 1200 °C by the use of residual stress relaxation or rapid cooling treatment^31^,^32^. Nevertheless, type II-IR gratings also have relatively larger insertion loss or larger cladding modes loss. For example, the researchers in Hong Kong Polytechnic University reported that the out-of-band insertion loss of type I-IR gratings was below 0.1 dB, whereas the insertion loss of type II-IR gratings was ranged from 0.6 dB to a few dB^30^. In 2012, Cook *et al.* reported on the production of regenerated FBGs using NIR femtosecond laser inscription and thermal regeneration^33^. The femtosecond-laser-induced regenerated FBGs can withstand temperatures up to 1200 °C, but have a low reflectivity of less than 8%. Recently, we reported on a highly polarization dependant phase-shifted FBG (P-PSFBG, i.e. Hi-Bi PS-FBG named in ref. 34) inscribed in a standard H_2_-free single-mode fibre by overexposure to a NIR femtosecond laser^34^. The spectral evolution from the original type I-IR FBG to the P-PSFBG was observed as a blueshift in the dip wavelength, *λ*_D_, and a rollover in the transmission loss at *λ*_D_. Negative index modulation was considered to be responsible for the formation of the P-PSFBG and could be employed to fabricate FBGs with excellent spectral properties as well as enhanced thermal stability. Moreover, to our knowledge, the negative-index type IIA gratings reported until now were all fabricated by UV laser exposure^10^,^11^,^12^,^13^,^14^,^15^,^16^,^17^,^18^, whereas the 800 nm NIR femtosecond laser has never been reported to successfully create such a negative-index grating formed by type IIA photosensitivity.

In this article, we demonstrate the producton of negative-index FBGs by NIR femtosecond laser overexposure and thermal regeneration. A positive-index type I-IR FBG was first inscribed, and then a P-PSFBG was formed by overexposure to a femtosecond laser. Subsequently, the P-PSFBG was annealed at 800 °C for 12 hours. Grating regeneration was observed during thermal annealing, and a negative-index FBG was obtained with a reflectivity of 99.22%, an insertion loss of 0.08 dB, a blueshift of 0.83 nm, and an operating temperature of up to 1000 °C for more than 10 hours. Further annealing tests showed that the thermal stability of the negative-index FBG was lower than that of a type II-IR FBG, but higher than that of a type I-IR FBG.

## Fabrication of the negative-index gratings

**Figure 1:**
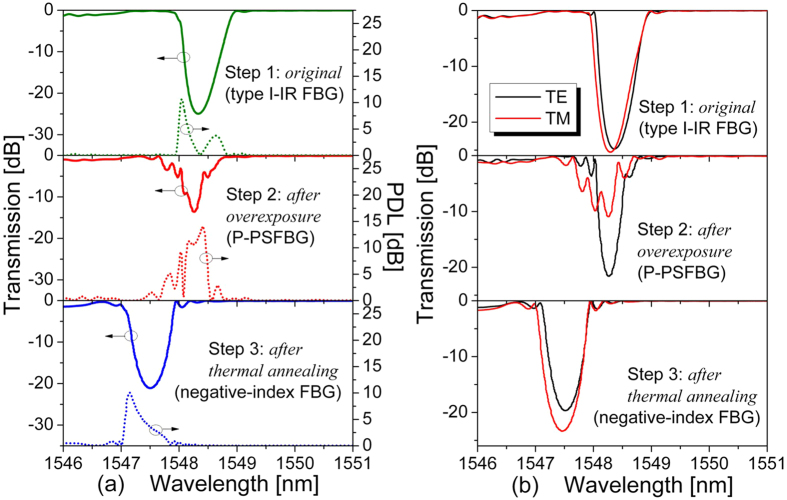
Transmission spectra of the original type I-IR fibre Bragg grating (FBG) (upper panel), highly polarization dependant phase-shifted FBG (P-PSFBG) (middle panel), and negative-index FBG (lower panel). (**a**) Transmission spectra (left axis) and polarization-dependent loss (PDL) (right axis). (**b**) Transmission spectra of two orthogonal polarization modes (TE, TM). All measurements conducted at room temperature, 20 °C.

The fabrication process of the negative-index FBG involved three steps, as shown in Fig. 1. In step 1, an ordinary positive-index type I-IR FBG was inscribed by a femtosecond laser directed through a phase mask. In step 2, the original type I-IR FBG was further exposed to a femtosecond laser with the same pulse energy, to form a P-PSFBG. In step 3, the P-PSFBG was annealed at 800 °C for 12 hours, and a negative-index FBG was finally obtained. The experiments and results in each step are discussed in detail next.

### Step 1: Fabrication of the positive-index type I-IR FBG by femtosecond laser inscription

The experimental setup used to fabricate type I-IR FBGs and P-PSFBGs is similar to that in our previous work^30^,^34^. Femtosecond laser pulses with a wavelength of 800 nm, a pulse-width of 100 fs, a repetition rate of 1 kHz, and a pulse energy of 4 mJ were generated by a Ti:sapphire regenerative amplifier system (Spectra-Physics, Solstice). The laser was linearly polarized with a 1/e^2^ Gaussian diameter of 6.2 mm. The pulse energy was attenuated by rotating a half wave-plate followed by a Glan polarizer. The laser beam was focused onto the fibre core using a cylindrical lens with a focal length of 50.2 mm through a uniform phase mask (Ibsen Photonics), which had a period of 1070 nm and a 0th-order diffraction of below 4%. A standard Corning SMF-28 single-mode fibre with the coating removed was fixed behind the phase mask at a distance of 300 μm. The focal width and Rayleigh length of the laser beam were calculated as 8.25 and 66.78 μm, respectively. The grating transmission spectra, polarization-dependent loss, and transmission spectra of two orthogonal polarization modes (TE, TM) were measured simultaneously by an optical component analyzer, which was composed of a tuneable laser (Agilent, 81940A), a polarization synthesizer (Agilent, N7786B), and an optical power meter (Agilent, N7744A).

In step 1, an ordinary type I-IR FBG with a transmission loss of −24.93 dB was inscribed in a H_2_-free single-mode fibre with a pulse energy of 240 μJ (i.e., a laser peak intensity of 9.0 × 10^12^ W/cm^2^) and an exposure time of 30 s. The measured results of grating transmission spectra, polarization-dependent loss (PDL), and transmission spectra of two orthogonal polarization modes (TE, TM) are shown in the upper panels of Fig. 1.

Moreover, the type I-IR FBG had a Gaussian-apodized index profile induced by the Gaussian laser beam with a 1/e^2^ diameter of 6.2 mm. The effective length of the FBG was defined by the full-width at half-maximum (FWHM) of the index profile^35^, and was calculated to be 3.65 mm. By using coupled-mode theory and transfer matrix method, the maximum index modulation in the type I-IR FBG was determined to be 1.3 × 10^−3^.

### Step 2: Fabrication of the P-PSFBG by overexposure to a femtosecond laser

**Figure 2:**
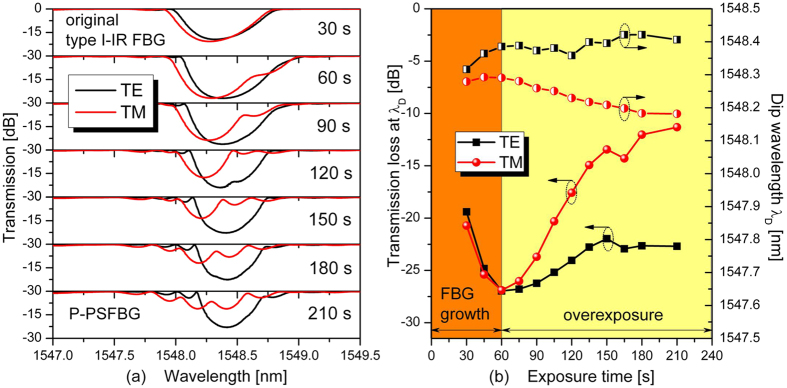
Transmission evolution of fibre Bragg grating (FBG) in case of overexposure. (**a**) Transmission spectrum evolutions of the two orthogonal polarization modes (TE and TM) from original type I-IR fibre Bragg grating (FBG) to highly polarization dependant phase-shifted FBG (P-PSFBG) in case of overexposure. (**b**) Measured transmission loss and Bragg wavelength of P-PSFBG as a function of exposure time.

In step 2, the original type I-IR FBG was further exposed to a femtosecond laser with the same pulse energy of 240 μJ for another 180 s. During this time, the grating degenerated gradually, as evidenced by the emergence and blueshift of phase-shifted peaks. As shown in Fig. 2, in the case of overexposure, the grating degeneration and the spectral evolution from the original type I-IR FBG to the PSFBG mainly occur in the TM polarization mode. For example, the transmission loss in the TM polarization mode decreases from −26.90 to −11.31 dB with a blueshift of about 0.11 nm, whereas the decrease of transmission loss is much smaller in the TE polarization mode (from −26.96 to −22.69 dB). As shown in the middle panels of Fig. 1, a P-PSFBG was obtained at the end of step 2 after a total exposure time of 210 s.

### Step 3: Fabrication of negative-index FBG by thermal annealing

In step 3, the thermal annealing was performed in a tube furnace (CARBOLITE MTF 12/38/ 250), which enabled temperatures as high as 1200 °C to be obtained. At first, the P-PSFBG was loosely placed in the furnace with no external stress applied. The temperature in close proximity to the grating was monitored using a thermocouple. Next, the temperature was raised from 20 °C to 800 °C in steps of 100 °C, with a heating rate of 100 °C/hour. After that, long-term annealing was performed by stabilizing the temperature at 800 °C for 12 hours. Finally, the temperature was gradually reduced from 800 °C to 20 °C.

**Figure 3:**
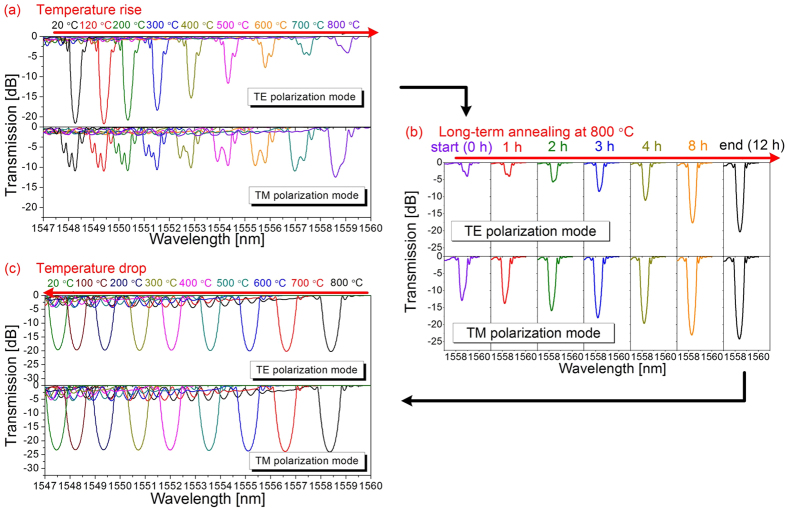
Transmission spectra evolution of the two orthogonal polarization modes (TE, TM) from highly polarization dependant phase-shifted fibre Bragg grating to negative-index fibre Bragg grating. (**a**) Temperature increase from 20 to 800 °C. (**b**) Long-term annealing at 800 °C for 12 hours. (**c**) Temperature reduction from 800 to 20 °C.

During the temperature increase, as shown in Fig. 3(a), the grating transmission dip in the TE polarization mode was gradually annealed out at temperatures above 400 °C (i.e., like a type I-IR grating), whereas spectral evolution from a PSFBG to an ordinary FBG was observed in the TM polarization mode without significant decay in the transmission dip. During the long-term annealing at 800 °C, as shown in Fig. 3(b), an abnormal regeneration could be observed progressively in both TE and TM polarization modes. During the temperature decrease, as shown in Fig. 3(c), the grating spectral shape and the transmission loss at *λ*_D_ almost remained unchanged.

**Figure 4:**
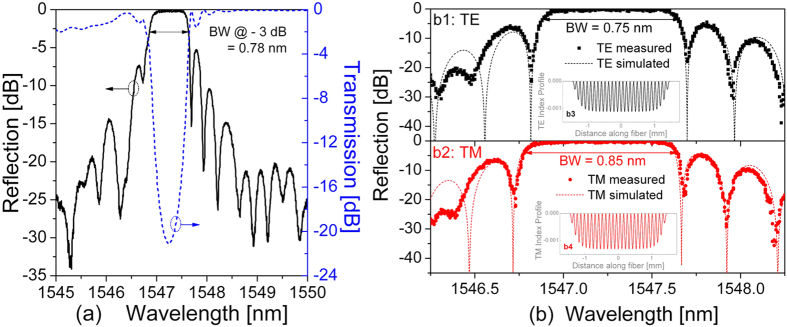
Spectra of the final negative-index FBG. (**a**) Measured reflection spectrum (left axis) and measured transmission spectrum (right axis). (**b**) Measured and simulated reflection spectra of two orthogonal polarization modes TE (b1) and TM (b2). BW, bandwidth. Inserts: index profile models used in simulation (b3: index profile model in TE polarization, b4: index profile model in TM polarization, the grating pitches are exaggerated 200 times for clarity).

The transmission spectra and the polarization-dependent loss of the final negative-index FBG are shown in the lower panels of Fig. 1, and the reflection spectra are shown in Fig. 4. It can be seen that the negative-index FBG has an excellent spectral shape with a transmission loss of −21.08 dB (i.e., a high reflectivity of 99.22%), an ultra-low insertion loss of 0.08 dB, a polarization-dependent loss below 10.05 dB, a 3 dB bandwidth of 0.78 nm, and a blueshift of 0.83 nm in *λ*_D_ compared with that of the original type I-IR FBG.

Furthermore, it can be seen from Fig. 1(b) that the final negative-index FBG has a symmetrical spectral shape in both TE and TM polarization modes, whereas the original type I-IR FBG has an asymmetrical spectral shape. The different symmetries in spectral shapes indicate that the original type I-IR FBG fabricated in Step 1 has a typical Gaussian index modulation profile, whereas the final negative-index FBG has a quasi-uniform index modulation profile due to the overexposure and thermal annealing applied in Steps 2 and 3. Moreover, the negative-index FBG has different transmission losses, of −19.68 and −23.36 dB, different central wavelengths, of 1547.51 and 1547.47 nm, and different 3 dB bandwidths, of 0.75 and 0.85 nm, respectively, in the TE and TM polarization modes.

Numerical simulations of the reflection spectra of the negative-index grating were carried out based on coupled-mode theory and transfer matrix method^34^. As shown in Fig. 4(b), quasi-uniform grating index profile models were employed in the simulations. The measured results were in good accordance with the simulated results, and the corresponding index modulation in the TE and TM polarization could be determined to be −1.1 × 10^−3^ and −1.3 × 10^−3^, respectively. Hence, the negative index modulation in the TM polarization mode was slightly larger than that in the TE polarization mode.

### Comparative annealing tests of the P-PSFBG and the original type I-IR FBG

For comparison, a similar annealing test was performed on the original type I-IR FBG, which had a transmission loss of −20.40 dB. The type I-IR FBG was inscribed in a H_2_-free fibre with the same pulse energy of 240 μJ as that previously used for fabricating the P-PSFBG, but with a much shorter exposure time (30 s for type I-IR FBG, without overexposure).

**Figure 5:**
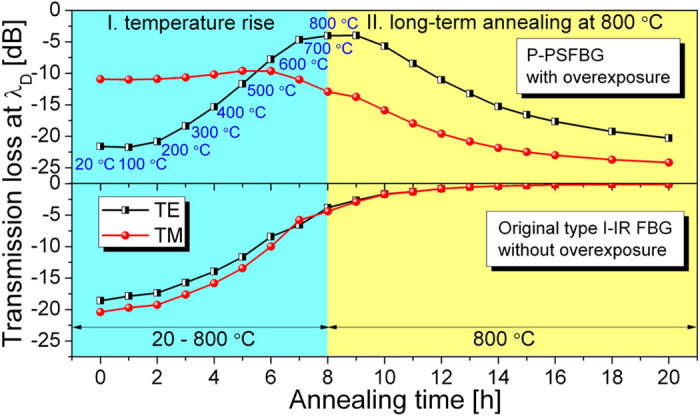
Evolution of transmission loss at dip wavelength, *λ*_D_ during thermal annealing. Upper panel: evolution of two orthogonal polarization modes (TE, TM) of the highly polarization dependant phase-shifted FBG (P-PSFBG). Lower panel: evoluation of original type I-IR fibre Bragg grating (FBG) during the temperature rise and the long-term annealing at 800 °C. The two gratings were inscribed with the same pulse energy of 240 μJ, but with different exposure times, 210 s and 30 s, respectively.

In the case of the P-PSFBG, as shown in Fig. 3 and in the upper panel of Fig. 5, thermal regeneration could be observed in both TE and TM polarization modes. Nevertheless, the transmission loss evolved differently in both TE and TM polarization modes of the P-PSFBG. For example, the transmission loss in the TE polarization mode decreased from −21.61 to −4.01 dB during the temperature rise and increased progressively from −4.01 to −20.29 dB after annealing at 800 °C for 12 hours, whereas the transmission loss in the TM polarization mode decreased slightly from −10.93 to −9.60 dB during the temperature rise and increased from −9.60 to −24.16 dB during the long-term annealing. Moreover, the regeneration in the TM polarization mode occurred earlier than that in the TE polarization mode.

In the case of the original type I-IR FBG, as shown in the lower panel of Fig. 5, the transmission loss in both the TE and TM polarization modes decayed gradually during annealing, and no thermal regeneration could be observed. After annealing, the grating was erased almost completely, with a transmission loss of −0.10 dB at *λ*_D_. Hence, the original FBG behaved like a typical type I-IR grating during the thermal annealing, and the negative-index FBG could not be created directly from the original type I-IR FBG.

Moreover, it should be noted that the annealing results of the original type I-IR FBG reported in this article are different from the previous results reported by Cook *et al.*^33^. In their work, thermal regeneration was observed in type I-IR FBGs under similar annealing conditions. The reason for the different annealing results relies on the fact that H_2_-loading was used in Cook’s work in fabricating the strong type I-IR FBGs^33^ and was reported to play an important role in grating regeneration^21^, whereas no H_2_-loading was used in fabricating the original type I-IR FBG in this article.

## Thermal stability of the negative-index gratings

### Short-term thermal stability of the negative-index FBGs

**Figure 6:**
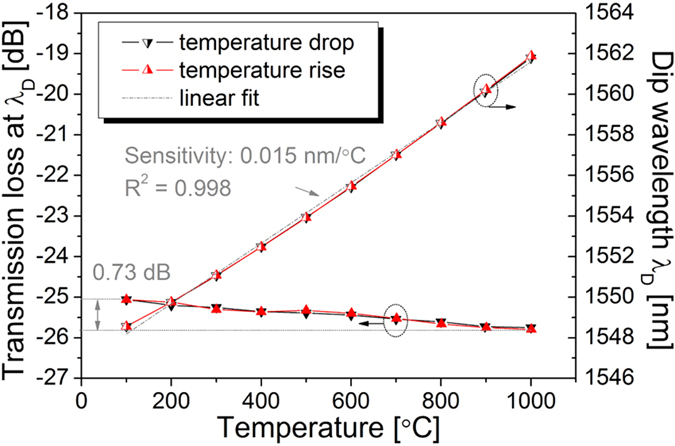
Negative-index FBG during short-term temperature cycling. Right axis: measured dip wavelength *λ*_D_. Left axis: transmission loss at *λ*_D_.

We investigated the short-term thermal stability of the negative-index FBGs via short-term temperature cycling. A negative-index FBG with a transmission loss of −25.01 dB and a dip wavelength of 1547.36 nm was created and placed in the furnace. The temperature was first raised from 100 to 1000 °C in steps of 100 °C, with a heating rate of 600 °C/hour, then stabilized at 1000 °C for 10 minutes, and finally reduced from 1000 to 100 °C in steps of 100 °C. As shown in Fig. 6, the dip wavelength *λ*_D_ shifted linearly with the temperature increase and decrease, with a temperature sensitivity of 0.015 nm/°C. Furthermore, the transmission loss at *λ*_D_ was slightly increased from −25.06 dB at 100 °C to −25.79 dB at 1000 °C, but returned to −25.05 dB when the temperature was cooled down to 100 °C. Hence, the dip wavelength and transmission loss of the negative-index FBG are repeatable with no hysteresis during short-term temperature cycling.

### Long-term thermal stability of the negative-index FBGs

**Figure 7:**
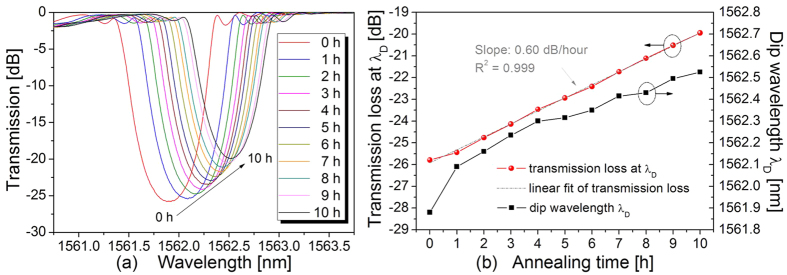
Long-term annealing study of the negative-index FBG at 1000 °C. (**a**) Transmission spectra evolution of the negative-index FBG at 1000 °C. (**b**) Measured dip wavelength *λ*_D_ (right axis) and transmission loss at *λ*_D_ (left axis) as a function of annealing time.

We also investigated the long-term thermal stability of the negative-index FBGs by annealing at 1000 °C for 10 hours. As shown in Fig. 7, the transmission loss at *λ*_D_ decayed slowly and almost linearly at a degeneration rate of 0.60 dB/hour (from −25.79 to −19.95 dB after 10 hours annealing at 1000 °C), and there was a redshift of 0.65 nm in the dip wavelength *λ*_D_. The redshift in *λ*_D_ during grating decay indicates that the average refractive index (d*n*_DC_) in the fibre core is increased when the index modulation (d*n*_AC_) is decreased. Hence, the value of d*n*_AC_ should be negative, and the tested FBG should be a negative-index grating.

### Comparison of long-term thermal stability of negative-index FBG and typical type II-IR FBG

**Figure 8:**
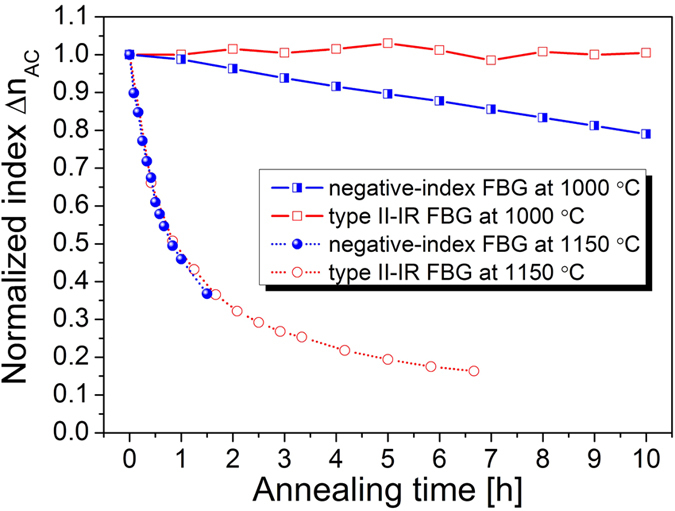
Evolution of normalized index modulation (Δn_AC_) of the negative-index fibre Bragg grating (FBG) and the typical type II-IR FBG during long-term annealing at 1000 °C and 1150 °C.

The long-term thermal stabilities of the negative-index FBG and the typical type II-IR FBG were compared. At first, a type II-IR FBG with a transmission loss of −22.43 dB and an out-of-band insertion loss of −2.18 dB was inscribed in a H_2_-free fibre with a pulse energy of 550 μJ (i.e., a laser peak intensity of 2.6 × 10^13^ W/cm^2^) and an exposure time of 25 s. Next, the type II-IR FBG and the negative-index FBG were annealed at the same temperature of 1000 °C for 10 hours, and subsequently annealed at a higher temperature of 1150 °C. The index modulation (d*n*_AC_) was calculated from the transmission loss and the grating bandwidth, and then normalized to its original value. The thermal stability of the FBGs was evaluated via the normalized index modulation (Δ*n*_AC_). As shown in Fig. 8, the thermal stability of the negative-index FBG was lower than that of the typical type II-IR FBG. For example, at 1000 °C, Δ*n*_AC_ of the type II-IR FBG was rather stable, whereas Δ*n*_AC_ of the negative-index FBG decayed slowly and almost linearly. At a higher temperature, 1150 °C, the value Δ*n*_AC_ of both the negative-index FBG and the type II-IR FBG decayed rapidly and exponentially, and the Δ*n*_AC_ were finally annealed out. The annealing results for the type II-IR FBG are in accordance with previous works^24^,^25^,^26^,^27^,^28^,^29^,^30^.

## Discussion

In the case of the original type I-IR FBG formed in step 1, as shown in Figs 1 and 5, a positive index modulation was induced by low-intensity NIR femtosecond laser pulses without overexposure, and erased by thermal annealing at high temperatures. The formation of such a type I-IR FBG is related to multiphoton nonlinear absorption process^25^. In the case of the P-PSFBG formed in step 2, as shown in Figs 1 and 2, the blueshift of the phase-shifted peak and the rollover in transmission loss at *λ*_D_ indicate that negative index modulation was induced in the fibre core by overexposure to the femtosecond laser^34^. Hence, the P-PSFBG should be a hybrid of a positive-index type I-IR grating and a negative-index grating^34^. During thermal annealing in step 3, as shown in Figs 3 and 5, the positive-index type I-IR grating should be erased gradually, along with an evolution to the characteristics of a negative-index grating, and hence lead to the spectral evolution from a PSFBG to a FBG and the thermal regeneration of a negative-index FBG.

Furthermore, anisotropic internal stress across the fibre core was created by overexposure to a femtosecond laser, and resulted in negative index modulation together with a localized high birefringence, owing to the photoelastic effect^34^,^36^,^37^. As a result, as shown in Figs 1 and 2, the negative index modulation within the P-PSFBG mainly existed in the TM polarization mode. During long-term thermal annealing at high temperatures, the internal stress within the P-PSFBG might be equilibrated and partially released^11^,^13^,^36^, leading to a reduction in birefringence and an increase in negative index modulation in the TE polarization mode. Consequently, as shown in Figs 3 and 5, grating regeneration could also be observed in the TE polarization mode after the grating regeneration occurred in the TM polarization mode, and, as shown in Figs 1 and 4, the negative-index FBG was fabricated successfully in both TE and TM polarization modes.

Moreover, the negative index modulation produced in the final negative-index FBG fabricated in step 3 could also be demonstrated by the blueshift of 0.83 nm in the Bragg wavelength of the negative-index FBG compared with that of the original type I-IR FBG (as shown in Fig. 1), and the redshift in the Bragg wavelength of the negative-index FBG during grating decay at 1000 °C (as shown in Fig. 7).

Grobnic *et al.* reported similar spectral evolutions in the growth and annealing of type II-IR FBGs^26^,^27^,^28^. During the fabrication of a type II-IR FBG, a type I-IR FBG was produced first, followed by erasure of the type I-IR FBG and finally regrowth to form a type II-IR FBG^28^. During the thermal annealing of a type II-IR FBG, the reflectivity increased first; this was considered an effect of erasing the type I-IR component^26^. Nevertheless, the negative-index FBGs presented in this article are different from typical type II-IR FBGs. Firstly, the negative-index FBG was inscribed with a much lower laser intensity than that used for inscribing a typical type II-IR FBG. In this study, we used a laser peak intensity of 9.0 × 10^12 ^W/cm^2 ^to inscribe the negative-index grating. The intensity was the same as that used for inscribing a type I-IR grating, and was far below the threshold of type II-IR grating formation^25^,^27^,^30^. Secondly, the spectral evolution in the fabrication of the negative-index FBG showed a strong polarization dependence, which has not been observed in the fabrication of type II-IR FBGs. Thirdly, the negative-index FBG showed an ultra-low insertion loss of 0.08 dB, which was comparable with that of a type I-IR FBG (typically below 0.1 dB) and much smaller than the damage-induced insertion loss within a type II-IR FBG (typically from 0.6 dB to a few dB)^25^,^27^,^30^. Finally, the negative-index FBG has a lower thermal stability than the typical type II-IR FBG. For example, the typical type II-IR FBG is quite stable at 1000 °C^25^,^26^,^27^,^28^,^29^,^30^, whereas the negative-index grating decayed slowly at the same temperature (as shown in Figs 7 and 8).

At an annealing temperature of 1000 °C, the negative-index FBG decays slowly, since the annealing temperature is slightly above the stress relaxation point of Ge-doped silica, i.e., ≈950 °C^11^. On the contrary, the type II-IR FBG appears to be quite durable at the same temperature, since it is formed by structural changes (i.e., damage) with a higher demarcation point. As a result, the photosensitivity of negative index modulation is of a mechanical nature and mostly related to internal stress^3^, and is similar to that in a UV-induced type IIA grating. Moreover, at a higher annealing temperature of 1150 °C, which is higher than the softening point, *T*_g_, of germanosilicate glass, both the negative-index FBG and the type II-IR FBG decay in the same way, since all types of defects are thermally faded out. Therefore, it could be inferred from our analysis that the formation of such a negative-index grating is most probably due to thermally regenerated type IIA photosensitivity.

## Conclusions

Negative-index FBGs were produced using femtosecond laser overexposure and thermal regeneration. At first, a positive-index type I-IR FBG was inscribed in a H_2_-free fibre using an 800 nm femtosecond laser through a phase mask, and then a P-PSFBG was fabricated from the type I-IR FBG through overexposure to a femtosecond laser. Subsequently, the P-PSFBG was annealed at 800 °C for 12 hours, during which time grating regeneration was observed. Finally, a negative-index FBG was obtained with a high reflectivity of 99.22%, an ultra-low insertion loss of 0.08 dB, a blueshift of 0.83 nm, and an operating temperature of up to 1000 °C for more than 10 hours. Further annealing tests showed that the thermal stability of the negative-index FBG was lower than that of a type II-IR FBG, but much higher than that of a type I-IR FBG. Hence, the formation of such a negative-index grating may result from thermally regenerated type IIA photosensitivity. Such a negative-index FBG could be used to develop a promising high-temperature sensor.

## Additional Information

**How to cite this article**: He, J. *et al.* Negative-index gratings formed by femtosecond laser overexposure and thermal regeneration. *Sci. Rep.* **6**, 23379; doi: 10.1038/srep23379 (2016).
